# Sirt3 deficiency does not affect venous thrombosis or NETosis despite mild elevation of intracellular ROS in platelets and neutrophils in mice

**DOI:** 10.1371/journal.pone.0188341

**Published:** 2017-12-13

**Authors:** Hideki Hayashi, Deya Cherpokova, Kimberly Martinod, Thilo Witsch, Siu Ling Wong, Maureen Gallant, Stephen M. Cifuni, Leonard P. Guarente, Denisa D. Wagner

**Affiliations:** 1 Program in Cellular and Molecular Medicine, Boston Children’s Hospital, Boston, Massachusetts, United States of America; 2 Department of Pediatrics, Harvard Medical School, Boston, Massachusetts, United States of America; 3 Department of Biology, Paul F. Glenn Center for the Science of Aging, Massachusetts Institute of Technology, Cambridge, Massachusetts, United States of America; 4 Division of Hematology/Oncology, Boston Children’s Hospital, Boston, Massachusetts, United States of America; Hospital for Sick Children, CANADA

## Abstract

Inflammation is a common denominator in chronic diseases of aging. Yet, how inflammation fuels these diseases remains unknown. Neutrophils are the primary leukocytes involved in the early phase of innate immunity and inflammation. As part of their anti-microbial defense, neutrophils form extracellular traps (NETs) by releasing decondensed chromatin lined with cytotoxic proteins. NETs have been shown to induce tissue injury and thrombosis. Here, we demonstrated that Sirt3, a nicotinamide adenine dinucleotide (NAD+)-dependent protein deacetylase, an enzyme linked to human longevity, was expressed in mouse neutrophils and platelets. Using Sirt3-/- mice as a model of accelerated aging, we investigated the effects of Sirt3 deficiency on NETosis and platelet function, aiming to detect enhancement of thrombosis. More mitochondrial reactive oxygen species (ROS) were generated in neutrophils and platelets of Sirt3-/- mice compared to WT, when stimulated with a low concentration of phorbol 12-myristate 13-acetate (PMA) and a high concentration of thrombin, respectively. There were no differences in *in vitro* NETosis, with or without stimulation. Platelet aggregation was mildly augmented in Sirt3-/- mice compared to WT mice, when stimulated with a low concentration of collagen. The effect of Sirt3 deficiency on platelet and neutrophil activation *in vivo* was examined by the venous thrombosis model of inferior vena cava stenosis. Elevation of plasma DNA concentration was observed after stenosis in both genotypes, but no difference was shown between the two genotypes. The systemic response to thrombosis was enhanced in Sirt3-/- mice with significantly elevated neutrophil count and reduced platelet count. However, no differences were observed in incidence of thrombus formation, thrombus weight and thrombin-antithrombin complex generation between WT and Sirt3-/- mice. We conclude that Sirt3 does not considerably impact NET formation, platelet function, or venous thrombosis in healthy young mice.

## Introduction

The aging population of the world is growing rapidly. Since aging is a general risk factor for many chronic conditions, there has been an increase in the incidence of cardiovascular disease [1, 2]. The cause of death has also changed from mainly infection and acute diseases, to degenerative and chronic diseases. It has been generally regarded that the imbalance of innate immunity and inflammation is an important etiological mechanism that causes age-related diseases [3].

Neutrophils are immune cells that play crucial roles in host defense and injury repair. In some conditions, neutrophils have detrimental effects in tissue injury and thrombosis through NETosis; cell death that releases decondensed chromatin structures [4–6]. We recently found that the susceptibility of neutrophils to form NETs increased with aging, and that peptidylarginine deiminase 4 (PAD4), an enzyme critical for NETosis, promotes thrombosis and age-related organ fibrosis [7, 8]. In general, there are several indicators which show that aging affects neutrophil function. Neutrophil counts [9, 10] and ROS generation [11] in neutrophils both increase with age in humans. These are implicated in predisposition toward NETosis as intracellular ROS production is essential for NETosis [12]. Currently, the etiology underlying neutrophil function that enhances age-related chronic disease remains unknown. To uncover the link between thrombosis, an age-related disease, and age-related alteration in neutrophil function, it is necessary to investigate aging-related genes such as Sirtuin 3; specifically, its role in NETosis, and its contribution to thrombus formation.

Platelets play crucial roles in thrombus development, and their function is also affected by aging [13]. Platelets from aged individuals readily aggregate in response to low concentrations of agonists [14]. Platelet activation, which releases the granular components platelet factor 4 (PF4) and beta-thromboglobulin (β-TG) was enhanced in aged individuals (age 70–98) compared to young individuals (age 12–40) [15]. Age-related susceptibility to arterial and venous thrombosis parallels enhanced platelet activation [16]. Therefore, using animal models of aging, it is important to address the etiology of age-related physiological alterations in platelets towards thrombosis.

Sirtuin family proteins are NAD+-dependent protein deacetylases, and have been long studied in animal models of aging. There are seven isoforms of mammalian Sirtuins, known as Sirtuin 1–7. Three of the seven isoforms (Sirt 3, 4, 5) are localized in mitochondria [17, 18]. Of these, Sirt3 is the only sirtuin that is linked to human longevity [19, 20]. Sirt3 regulates activity of metabolic and respiratory enzymes in mitochondria by deacetylation of acetylated residues on target enzymes [18]. The main function of Sirt3 is to down-regulate mitochondrial oxidative stress [21] by activation of manganese super oxide dismutase (MnSOD), alternatively called superoxide dismutase 2 (SOD2), one of several ROS degrading enzymes [22]. Reduction of Sirt3 levels is one of the possible mechanisms that links age-related disease and enhanced susceptibility to oxidative stress observed in aged cells, such as mesenchymal stromal cells and cells of the auditory cortex [23, 24]. Sirt3 deficiency accelerates phenotypes observed in aging, such as spontaneous carcinogenicity [25–27] and elevation of intracellular ROS levels [28]. Thus, Sirt3-/- mice can be considered as an animal model of accelerated aging [29].

We hypothesized that age-related decline of Sirt3 activity leading to elevation of intracellular ROS could induce functional alteration in neutrophils and platelets, resulting in inflammation-driven disorders. In this study, we investigated the expression of Sirt3 in these blood cells and the effects of Sirt3 deficiency on neutrophil and platelet function, aiming to detect enhancement of thrombosis.

## Materials and methods

### Study approvals and animals

All experimental procedures involving animals were reviewed and approved by the Institutional Animal Care and Use Committee of Boston Children’s Hospital (protocol numbers: 14-03-2631R, 14-02-2609R and 17-01-3308R). All methods were performed in accordance with the approved guidelines and regulations. All surgery was conducted under isoflurane anesthesia, and all efforts were made to minimize suffering. All mice received food and water *ad libitum* and daily health checks. Mice with constitutive Sirt3 deficiency were originally generated by Dr. Frederick Alt [18]. Heterozygous male Sirt3 mice (Sirt3+/-) were backcrossed to C57BL/6J (Jackson Laboratory, Bar Harbor ME) for at least 12 generations. In this study mice used were between 9 and 12 weeks old.

### Basic blood parameters

Blood was collected via the retro-orbital sinus into EDTA-coated capillary tubes under isoflurane anesthesia. Complete differential blood cell counts were determined using a Hemavet 950FS Veterinary Multispecies Hematology System (Drew Scientific).

### Neutrophil isolation for *in vitro* neutrophil experiments

Neutrophils were isolated from peripheral blood [30]. Briefly, blood was collected in PBS containing 1% (wt/vol) BSA and 15 mM EDTA. After centrifugation, blood cells were resuspended and layered onto Percoll gradients of 78%, 69% and 52% in PBS, centrifuged and cells at the 69%/78% interface were collected. Red blood cells were lysed by the addition of a hypotonic solution. Cell count was determined on a hemocytometer. Neutrophil purity was established to be >90%, as assessed by Wright–Giemsa staining following cytospin.

### Preparation of washed platelets and platelet aggregation study

Murine blood was collected in tubes containing 0.2 μg/ml enoxaparin. Platelet-rich plasma (PRP) was obtained by two centrifugation cycles of 300 × *g* for 7 min at RT. Aggregation following ADP stimulation was analyzed in PRP. All other measurements were carried out with washed platelets. For this, PRP was pelleted at 700 × *g* in the presence of prostacyclin (PGI_2_) (0.1 μg/ml) and apyrase (0.02 U/ml). The platelet pellet was washed twice in modified Tyrode-HEPES buffer (134 mM NaCl, 0.34 mM Na_2_HPO_4_, 2.9 mM KCl, 12 mM NaHCO_3_, 5 mM HEPES, 1 mM MgCl_2_, 5 mM glucose, 0.35% BSA, pH 7.4) containing PGI_2_ and apyrase. Platelet suspensions (a total of 6 × 10^7^ platelets) in Tyrode-HEPES buffer containing 2 mM CaCl_2_ were stimulated with the indicated agonists and light transmission was recorded on a Chronolog platelet aggregometer. The number of animals used for this study was as follows: collagen induced aggregation, WT n = 11, Sirt3-/- n = 9. ADP induced aggregation, WT n = 10, Sirt3-/- n = 7, thrombin induced aggregation, WT n = 7, Sirt3-/- n = 7.

### Measurement of serotonin release

Washed platelets were prepared as described above. Platelet suspension (120 μl with 5 x 10^5^ platelets/μl) in Tyrode’s-HEPES buffer containing 1 mM CaCl_2_ was stimulated with the indicated agonists for 5 min at 37°C and constantly shaken at 400 rpm (Eppendorf Thermomixer). Supernatant was collected after a brief centrifugation step at 700 × *g* and cell debris removed by centrifugation at 21,950 × *g* for 5 min at 4°C. Total serotonin content was measured in platelet lysates. Serotonin levels in supernatants or platelet lysates were determined using Serotonin ELISA ^Fast Track^ (LDN GmbH & Co.KG, Nordhorn, Germany) according to the manufacturer’s instructions. The number of animals used for this study was as follows: unstimulated, collagen- or thrombin-activated platelets: WT n = 4, Sirt3-/- n = 4; total platelet serotonin content: WT n = 3, Sirt3-/- n = 4.

### Western blot analysis

Isolated neutrophils or washed platelets were homogenized in ice-cold RIPA buffer supplemented with protease inhibitor cocktail (Sigma). Proteins were resolved on a 4–20% SDS/PAGE gel and electroblotted onto PVDF membranes using an iBlot Gel Transfer Device (Thermo Fisher Scientific). The membranes were blocked with 5% BSA in TBS/0.05% Tween-20 for 2 hrs at room temperature and incubated overnight at 4°C with rabbit monoclonal anti-Sirt3 (Cell Signaling, #5490, 1:500). Membranes were then probed with a horseradish peroxidase–conjugated anti-rabbit IgG secondary antibody (Bio-Rad Laboratories, 1:4000). Detection was carried out with a Pierce ECL Western Blotting substrate (Thermo Fisher Scientific). Equal loading was confirmed by probing for anti-GAPDH mouse monoclonal antibody (Ambion, #AM4300, 1:20,000) or anti-β-Tubulin rabbit monoclonal antibody (Cell Signaling, #5346, 1:1000).

### *In vitro* NETosis assays

Isolated neutrophils from peripheral blood were resuspended in RPMI/HEPES and were allowed to adhere at 37°C, 5% CO_2_ to plastic plates (CellBIND, Corning) for 20 min before stimulation with ionomycin (4 μM) for 2 hr or phorbol 12-myristate 13-acetate (PMA) (25 nM, 100 nM) for 4 hr. After incubation, cells were fixed in 2% (vol/vol) paraformaldehyde followed by DNA staining with Hoechst-33342 (Thermo Fisher Scientific). Fluorescent images were acquired using an Axiovert 200M widefield fluorescence microscope (Zeiss) in conjunction with an Axiocam MR3 monochromatic CCD camera (Zeiss) and analyzed with Zeiss Axiovision software. NETs were counted from eight different fields per well in duplicates and expressed as percentages of NET-forming cells out of the total number of cells per well. The number of animals used for this study was as follows: WT n = 7, Sirt3-/- n = 7.

### Flow cytometry for neutrophil activation

Analysis of reactive oxygen species (ROS) formation was performed using flow cytometry (BD, FACS CantoII) and analyzed using FlowJo software version 8.8.7 (Tree Star Inc., Ashland, OR). Whole blood was collected using heparin-coated capillary tubes via the retro-orbital venous sinus and stimulated with ionomycin or PMA for 30 min at 37°C in the presence of dihydrorhodamine-123 (Thermo Fisher Scientific) for cytosolic ROS and MitoSox^TM^Red (Thermo Fisher Scientific) for mitochondrial ROS[31]. After stimulation, red blood cell lysis, and several washing steps, cells were stained using Alexa Fluor 647-conjugated anti-mouse Ly-6G monoclonal antibody (BioLegend, #127610, 1:300). Neutrophils were gated by forward-and-side scatter and positive signal for Ly6G. Rhodamine positive or PE positive neutrophils were quantified for ROS production. The number of animals used for this study was as follows: WT n = 7, Sirt3-/- n = 5.

### Flow cytometry for platelet activation

Washed platelets (2.0 × 10^4^/μL) were stimulated with 100 mU/mL thrombin for 20 min in the presence of dihydrorhodamine-123 for cytosolic ROS and MitoSox^TM^Red for mitochondrial ROS. After stimulation, cells were stained using APC-conjugated anti-mouse CD41 monoclonal antibody (BioLegend, #133914, 1:100). Platelets were gated by forward-and-side scatter and positive signal for CD41. Rhodamine positive or PE positive cells were quantified for ROS production. The number of animals used for this study was as follows: WT n = 6, Sirt3-/- n = 6.

### Measurement of plasma DNA, thrombin anti-thrombin (TAT) complexes and soluble P-selectin

Blood was collected into 1/10 final volume of 3.2% (wt/vol) sodium citrate anticoagulant. After centrifugation of whole blood at 3300 × *g* for 5 min, plasma was collected and centrifuged at 16100 × *g* for 5 min to remove remaining cellular components. Plasma DNA was measured using the Quant-iT PicoGreen dsDNA Assay Kit (Thermo Fisher Scientific, P7589). TAT complexes (Abcam, ab137994) and soluble P-selectin (R&D systems, MPS00) were measured by ELISA according to the manufacturer’s instructions.

### Venous stenosis model of deep vein thrombosis

Mice were anesthetized with 3.5% isoflurane and anesthesia was maintained at 2% isoflurane in 100% oxygen. A midline laparotomy was performed and the inferior vena cava was exposed. Side branches between the renal and iliac veins were ligated with 7/0 polypropylene suture. A 30-G spacer was placed parallel to the inferior vena cava and 7/0 polypropylene suture was used to partially ligate the inferior vena cava (IVC) to ~10% of its original diameter [32]. The spacer was then removed and the mouse was sutured and allowed to recover. After 3 hr, mice were anesthetized with isoflurane, blood was collected via the retro-orbital venous sinus followed by sacrifice and the IVCs were exposed to allow for collection of the IVC vessel wall or thrombi formed within the IVC. Thrombus length was measured and thrombi were embedded in optimal cutting temperature (OCT) compound for cryosectioning. The number of animals used for this study was as follows: WT n = 12, Sirt3-/- n = 11.

### Statistics

Data are presented as mean ± SEM and analyzed using Student’s t tests unless otherwise noted. For the analysis of thrombus frequencies, χ2 tests of contingency tables were used. P<0.05 was considered significant.

## Results

### Sirtuin 3 is expressed in neutrophils and intracellular ROS was higher in neutrophils isolated from Sirt3-/- mice compared to WT mice

**Fig 1:**
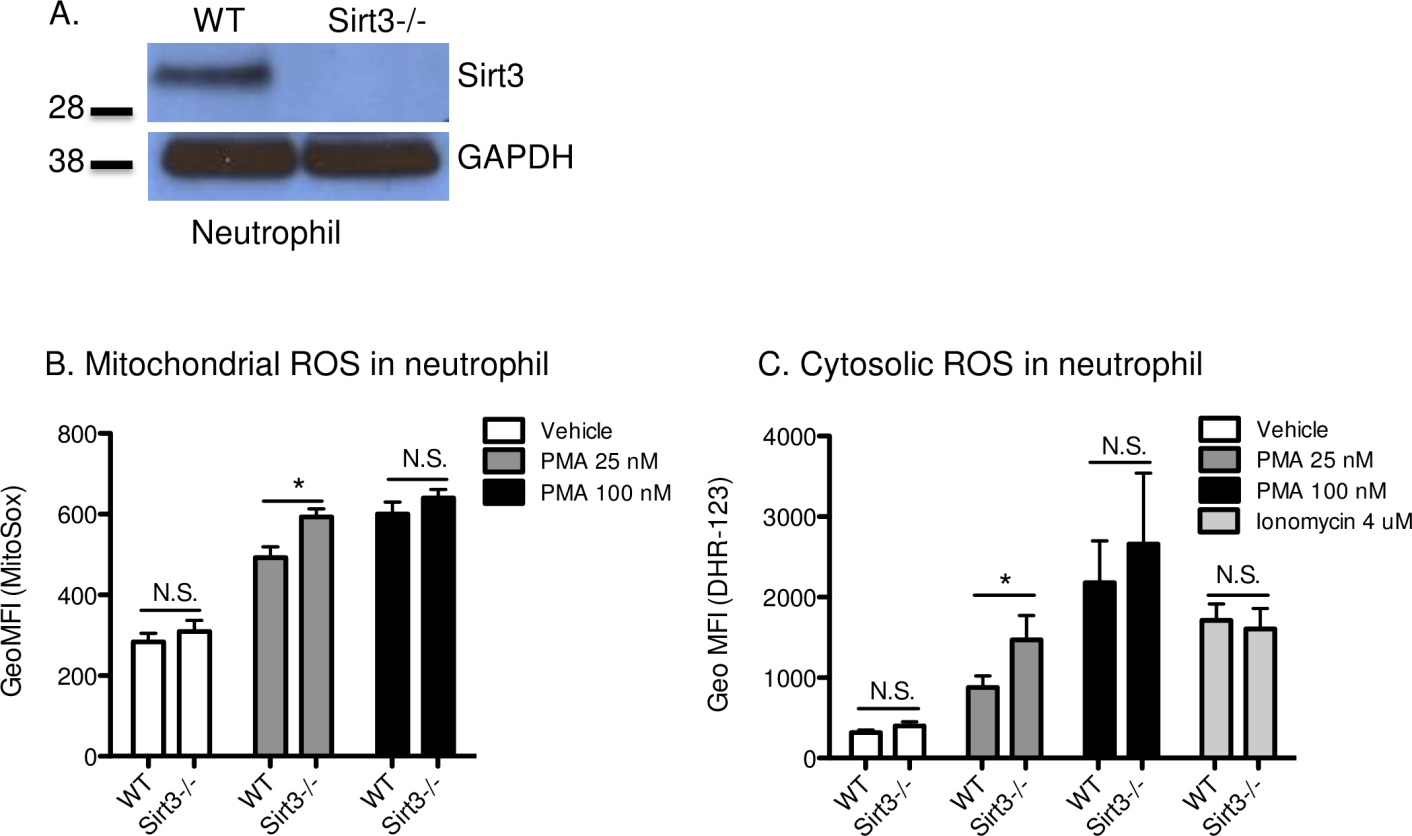
Deficiency of Sirt3 augmented production of ROS in neutrophils. (A) Western blot analysis using bone marrow neutrophil lysates from Sirt3-/- mice or WT mice. Photograph of a representative blot is shown. (B) and (C) represent flow cytometric determination of mitochondrial and cytosolic ROS in neutrophils. Diluted anti-coagulated whole blood from Sirt3-/- mice and WT mice was incubated with (B), (C) PMA (25, 100 nM), (C) ionomycin (4 μM) or vehicle for 30 min at 37°C in the presence of (B) MitoSox or (C) dihydrorhodamine-123. MitoSox or Rhodamine-positive neutrophils were quantified by flow cytometry for each condition. n = 5–7. *P<0.05 vs WT (Student’s t tests).

Sirt3 is a protein deacetylase that is specifically localized in mitochondria and contributes to regulation of intracellular ROS. Despite the importance of ROS in neutrophil function, Sirt3 expression in these cells has not been studied. Western blot analysis of isolated bone marrow neutrophils revealed that Sirt3 was present in neutrophils from WT mice, but not from Sirt3-/- mice (Fig 1A). When we observed expression of Sirt3 in neutrophils, an investigation was launched to compare the fundamental functions of neutrophils from Sirt3-/- and WT mice.

Elevation of intracellular ROS is an essential factor for the anti-microbial activity of neutrophils [33], such as during phagocytosis [34] and NETosis [12]. Sirt3 regulates both production and degradation of ROS in mitochondria [21]. For the degradation system of mitochondrial ROS, MnSOD plays a central role, and its activity is directly regulated by Sirt3 [22]. Thus, Sirt3 deficiency was hypothesized to result in continuous inactivation of MnSOD, followed by incomplete degradation of ROS. The effect of Sirt3 deficiency on the levels of mitochondrial ROS in neutrophils was examined using the specific indicator, MitoSox. Under static conditions, there were no differences in mitochondrial ROS between neutrophils isolated from Sirt3-/- and WT mice (Fig 1B). When neutrophils were stimulated with PMA at the concentration of 25 nM, higher ROS levels were observed in neutrophils from Sirt3-/- mice compared to those from WT, indicating that Sirt3-/- neutrophils are more sensitive to low grade stimulation. At 100 nM, however, elevation of ROS levels in both strains reached similar levels (Fig 1B). Higher cytosolic ROS, determined by dihydrorhodamine 123, were also observed in Sirt3-/- mice at 25 nM and not at 100 nM PMA (Fig 1C). The calcium ionophore ionomycin activates NADPH oxidase [35, 36], thus cytosolic ROS in neutrophils was elevated (Fig 1C). However, the elevation was similar in WT and Sirt3-/- neutrophils.

### Sirt3-/- and WT neutrophils showed no difference in *in vitro* NETosis

**Fig 2:**
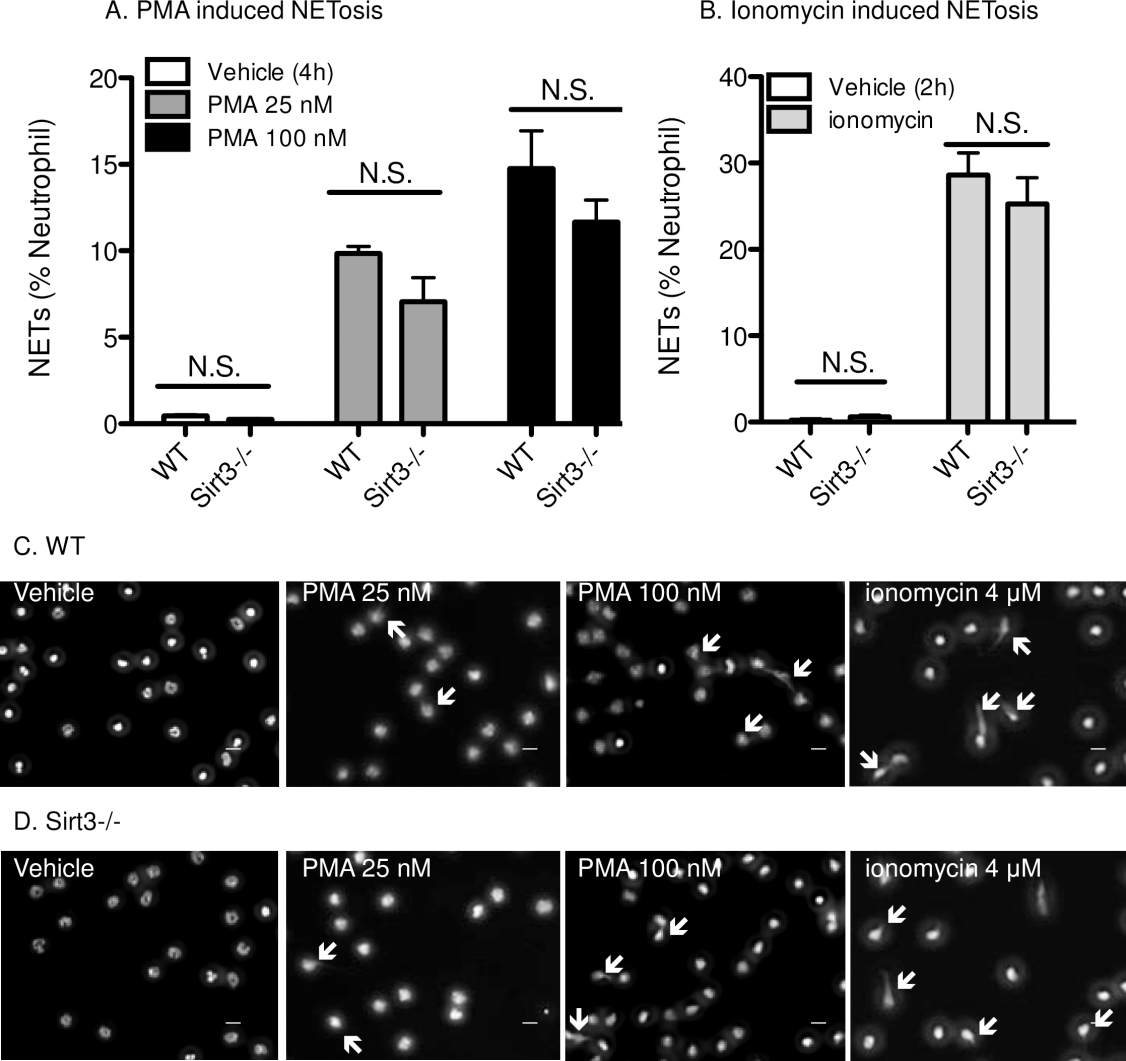
No difference was observed in *in vitro* NETosis between Sirt3-/- and WT. Peripheral neutrophils isolated from Sirt3-/- mice and WT mice were stimulated with (A) PMA (25, 100 nM, 4h) or (B) ionomycin (4 μM, 2h). The percentage of neutrophil extracellular traps (NET) generation was evaluated. n = 7, *P<0.05 vs WT (Student’s t tests). Fluorescent images of NET formation after PMA (25, 100 nM, 4h) or ionomycin (4 μM, 2h) stimulation and Hoechst staining of peripheral neutrophils isolated from (C) WT mice and (D) Sirt3-/- mice (Scale bar: 20 μm). Arrows indicate NETs.

ROS is one of the key mediators for NETosis, and its elevation induces it [12]. We speculated that neutrophils from Sirt3-/- mice would be predisposed to NETosis. Therefore, we investigated whether higher susceptibility to ROS generation in neutrophils from Sirt3-/- mice would prime them for NETosis. Peripheral neutrophils isolated from Sirt3-/- and WT mice were incubated with either PMA, a ROS-dependent inducer of NETosis, or ionomycin, a ROS-independent inducer of NETosis, for the indicated times. The formation of NETs was quantified by microscopy (Fig 2). Stimulation with PMA (Fig 2A, 2C and 2D) or ionomycin (Fig 2B, C, D) resulted in similar percentages of NETting neutrophils isolated from WT (Fig 2C) and Sirt3-/- mice (Fig 2D), indicating that Sirt3 deficiency did not impact NETosis susceptibility.

### Deficiency of Sirt3 in platelets increased mitochondrial ROS production

**Fig 3:**
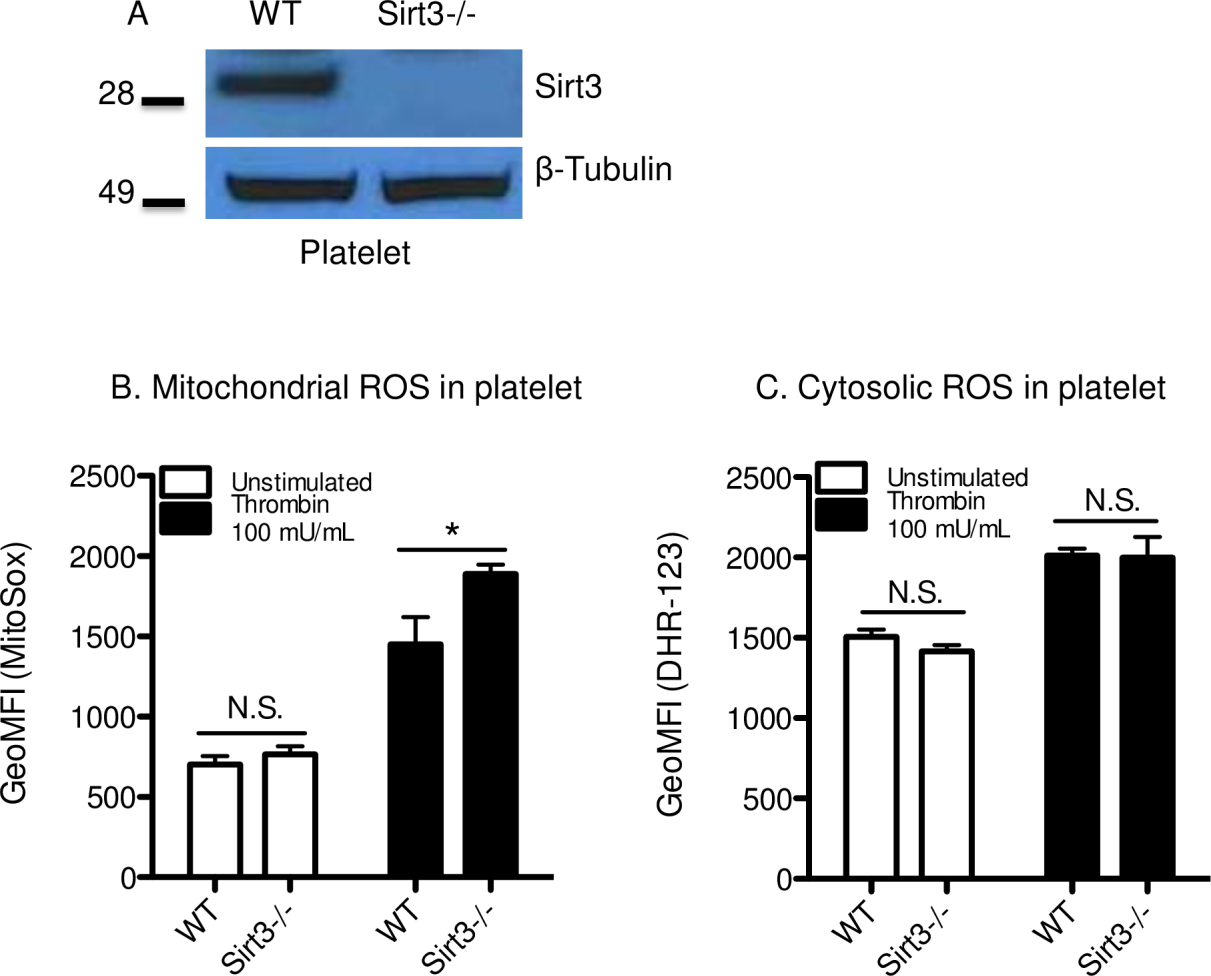
Deficiency of Sirt3 in platelets increased mitochondrial ROS production. (A) Western blot analysis using washed platelets from Sirt3-/- mice or WT mice. Photograph of a representative blot of experiments is shown. (B), (C) Flow cytometric determination of mitochondrial and cytosolic ROS in platelets. Washed platelets were incubated with thrombin (100 mU/mL) or vehicle for 30 min at 37°C in the presence of (B) MitoSox or (C) dihydrorhodamine-123. MitoSox or Rhodamine positive platelets were quantified by flow cytometry for each condition. n = 6, *P<0.05 vs WT (Student’s t tests).

Western blot analysis confirmed that Sirt3 was expressed in mouse platelets (Fig 3A), as was reported recently in human platelets [37]. Similar levels of major platelet glycoproteins were expressed in the platelets from WT and Sirt3-/- mice (Table 1). Mitochondria in platelets generate ATP as well as ROS [38]. ROS is a critical mediator in the process of platelet activation, including α-granule secretion, CD40L surface expression and GPIbα shedding [39–41]. We speculated that Sirt3 deficiency in platelets would result in excess ROS accumulation. When platelets were stimulated with thrombin 100 mU/mL, higher levels of mitochondrial ROS were observed in platelets isolated from Sirt3-/- mice compared with WT mice (Fig 3B). There were no differences between both genotypes in cytosolic ROS levels (Fig 3C).

**Table 1:** Expression levels of platelet surface glycoproteins.

	WT		Sirt3-/-	
	Mean	SEM	Mean	SEM
**GPIb**	100.0	2.4	97.9	3.5
**GPV**	100.0	1.8	100.5	0.3
**GPIX**	100.0	1.4	105.1	3.5
**GPVI**	100.0	1.1	99.0	1.5

Washed platelets from both genotypes were stained with antibodies to surface glycoproteins and analyzed by flow cytometer. Expression levels of each glycoprotein in Sirt3-/- platelets were shown as relative values to that of WT mice using geometric mean fluorescence intensity. n = 4. Statistically, no differences were observed between two genotypes.

### Sirt3 deficiency had only a minor impact on platelet functions

**Fig 4:**
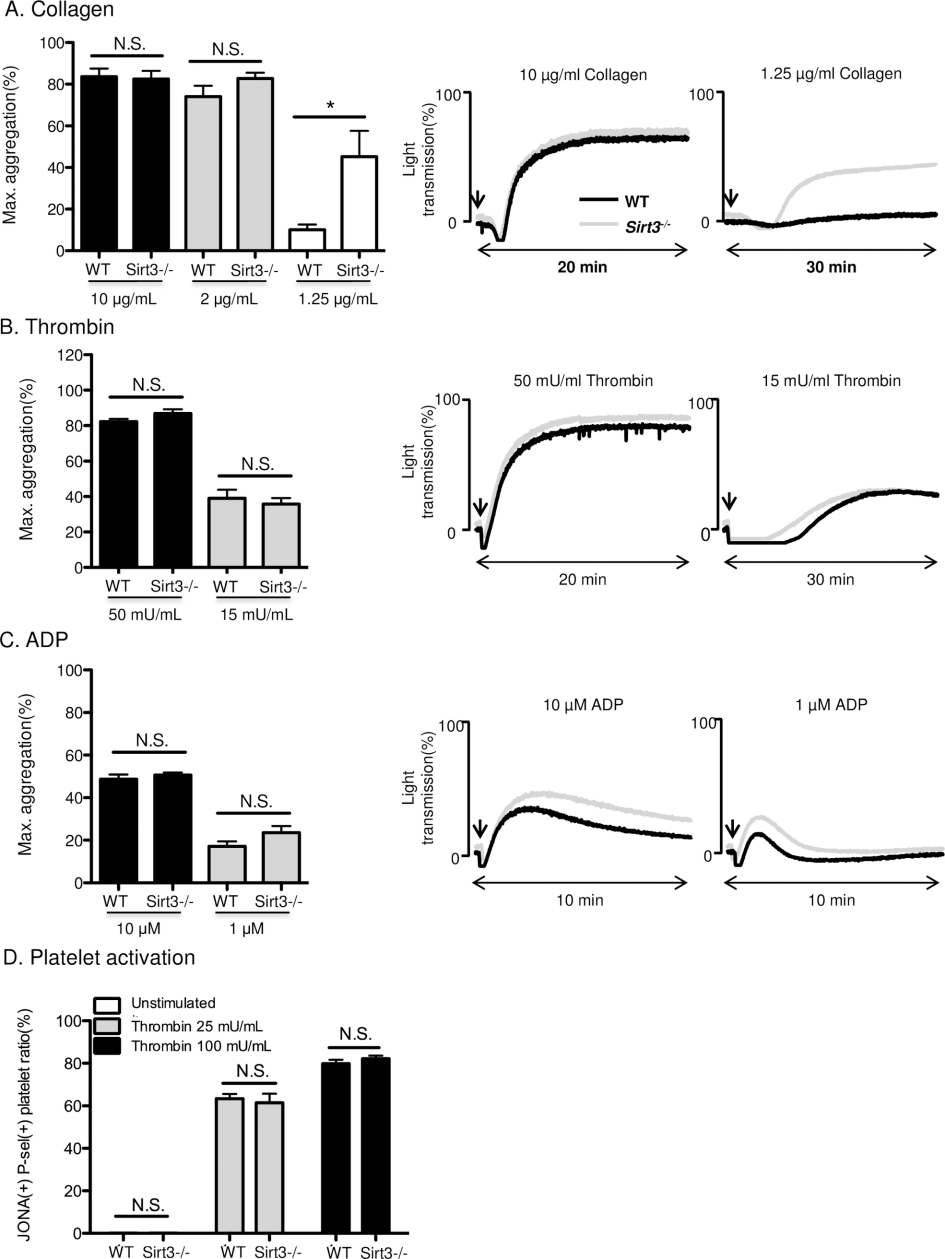
Sirt3 deficiency had a minor impact on platelet functions. Platelet aggregation was induced by (A) collagen, (B) thrombin and (C) ADP at the concentration described using (A), (B) washed platelets or (C) platelet-rich plasma (PRP) from Sirt3-/- mice and WT mice. Representative aggregation traces are on the right. n = 7–10. (D) Washed platelets from Sirt3-/- mice and WT mice were incubated with thrombin 25 or 100 mU/mL for 20 min at 37°C. Percentage of JON/A and P-selectin double positive cells were quantified by flow cytometry. n = 4.*P<0.05 vs WT (Student’s t tests).

Next, we investigated the effect of Sirt3 deficiency on platelet functions. Platelet aggregation was induced by three different types of agonists; collagen, thrombin and ADP (Fig 4). When platelets were stimulated with a low concentration of collagen, aggregation was significantly augmented in platelets from Sirt3-/-, compared with those from WT mice (Fig 4A). By contrast, low concentration of collagen induced similar platelet dense granule release in WT and Sirt3-deficient platelets, as determined by the measurement of serotonin release in the supernatant of agonist-stimulated platelets (S2 Fig). There were no significant differences in platelet aggregation induced by thrombin and ADP (Fig 4B and 4C) nor in thrombin-induced surface exposure of P-selectin and activation of GPIIbШa as determined using flow cytometry (Fig 4D).

### Venous thrombus formation was similar between Sirt3-/- and WT mice

**Fig 5:**
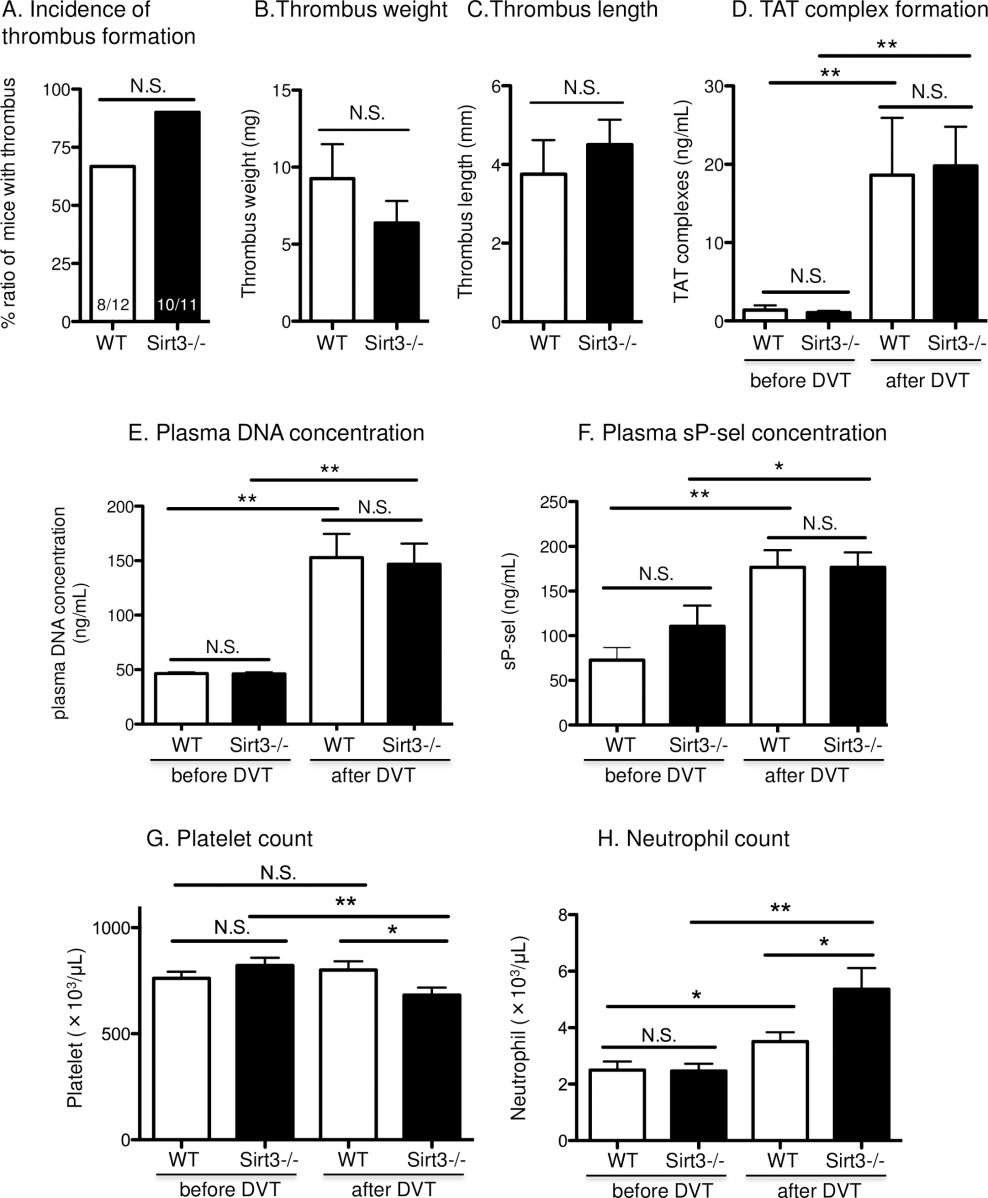
Deficiency of Sirt3 did not affect venous thrombosis *in vivo*. Sirt3-/- mice and WT mice underwent inferior vena cava (IVC) stenosis for 3 hrs. (A) Incidence of thrombus formation, (B) weight and (C) length are presented. (D) Thrombin anti-thrombin (TAT) complex, (E) plasma DNA concentration or (F) plasma soluble P-selectin concentration were determined. Circulating (G) platelet and (H) neutrophil counts were evaluated before (0 hr) and after (3 hrs) IVC stenosis. n = 11–12, *P<0.05, **P<0.01 vs WT or corresponding group (Student’s t tests). Thrombus frequencies were analyzed using χ2 tests of contingency tables.

Next we examined the effect of Sirt3 deficiency on platelet and neutrophil activation by physiological agonists *in vivo*, and whether the deficiency enhances venous thrombosis. Sirt3-/- and WT mice were subjected to inferior vena cava (IVC) stenosis, which recapitulates key features of deep vein thrombosis (DVT) progression on humans [42]. In this model, platelets and NETosis are indispensable for DVT initiation and propagation [7, 32, 42]. Stenosis with 90% reduction of blood flow was done for 3 hrs in the inferior vena cava (IVC). This resulted in the formation of thrombi in 67% of WT mice (8 out of 12) and 90% of Sirt3-/- mice (10 out of 11) (Fig 5A). However, there were no significant differences in the incidence of thrombus formation, thrombus weight, or length between Sirt3-/- and WT mice (Fig 5A–5C). As a marker of pro-coagulant state, the concentration of thrombin-antithrombin (TAT) complexes in plasma was evaluated, and no differences between the two genotypes were found (Fig 5D). Elevation of plasma DNA concentration implicates possible NETs formation *in vivo*. Three hour-stenosis profoundly increased plasma DNA concentrations in both genotypes, but there was no difference between the two genotypes (Fig 5E) in agreement with our *in vitro* NETosis results (Fig 2).

In this DVT model, P-selectin released from endothelial Weibel-Palade bodies upon cell activation plays a crucial role in the recruitment of leukocytes and development of thrombus formation [42]. Soluble P-selectin levels are considered to be an indicator of activation for both endothelium and platelets. Therefore, the soluble form of P-selectin was quantified (Fig 5F). However, there was no significant difference between the two genotypes. We previously reported that a reduction of the circulating platelet counts, due to platelet adhesion to endothelial von Willebrand factor, was induced by stenosis [32]. Therefore, platelet consumption in the thrombus could be considered a response to thrombus formation. To examine this response, circulating blood cell counts were monitored before and after stenosis. A significant reduction in circulating platelets at this early time point (3 hrs) was observed only in Sirt3-/- mice (Fig 5G). Neutrophil count was elevated after DVT formation in both genotypes, and significantly higher numbers were observed in Sirt3-/- compared with WT mice (Fig 5H), indicating that the systemic response to thrombosis was enhanced in Sirt3-/- mice.

## Discussion

In this study, we discovered that Sirt3 is expressed in neutrophils, and that Sirt3 deficiency increases neutrophil intracellular ROS levels after stimulation. Deficiency of Sirt3 in neutrophils resulted in elevation of ROS concentration both in mitochondria and the cytosol, but it did not affect NET formation. We also confirmed that Sirt3 is expressed in platelets and showed that its deficiency resulted in elevation of mitochondrial ROS concentration in platelets. Sirt3 deficiency augmented platelet aggregation induced by a low concentration of collagen, however, it did not alter aggregation induced by a higher concentration of collagen, or other stimulants. Thrombus formation *in vivo* was not significantly affected by Sirt3 deficiency.

In resting neutrophils and platelets, both mitochondrial and cytosolic ROS levels were comparable in the two genotypes (Figs 1 and 3). Under mild stimulation, slight elevation of intracellular ROS was observed in neutrophils and platelets from Sirt3-/- compared with WT. These results are consistent with previous reports showing elevation of superoxide levels in mouse embryonic fibroblast (MEFs) or human mesenchymal stromal cell (hMSC) with reduced Sirt3 levels that were observed only in response to exogenous stress [23, 25]. In the static condition, mitochondrial ROS levels are strictly regulated and kept low by MnSOD [43]. The mechanism through which this happens is still unclear. Sirt3 may function as a stress sensor [44] that is activated only in response to elevated intracellular oxidative stress.

In neutrophils under stimulated conditions, both mitochondrial and cytosolic ROS were higher in Sirt3-/- compared with WT (Fig 1B and 1C). Similar observations were made with an inhibitor of mitochondrial cytochrome bc1 complex, which elevated mitochondrial ROS and led to activation of NADPH oxidase (NOX) followed by elevation of intracellular ROS [45]. Therefore, the higher concentration of mitochondrial ROS that we observed might trigger activation of NOX resulting in elevation of cytosolic ROS. Overexpression of Sirt3 attenuates intracellular ROS production, which leads to beneficial effects on inflammation, cellular senescence, and age-associated tissue fibrosis [28, 46–49]. It would be of great interest to examine alterations of neutrophil function in Sirt3 overexpressing transgenic mice, as NETosis in these mice could be significantly inhibited. It is possible that under high stress conditions, such as those found in sepsis and trauma [50], Sirt3 may become upregulated and its deficiency would show higher negative impact on the host. This possibility requires further investigation.

We recently reported that PAD4 deficiency, followed by reduced NETosis, ameliorates the effects of age-related fibrosis [8]. Because Sirt3 deficiency in mice is regarded as an aging model, it was speculated that neutrophils from Sirt3-/- mice would be predisposed to NETosis, due to a larger amount of intracellular ROS. Here, higher concentrations of ROS were observed under mild stimulation, and subtle increase may not be enough to enhance NETosis. Sirt3 deficiency resulted in higher mitochondrial ROS level in platelets with thrombin stimulation (Fig 3B). However, cytosolic ROS levels in platelets from Sirt3-/- mice were unaltered (Fig 3C). According to a previous report, intracellular ROS in platelets were mainly generated by NADPH oxidase, and the contribution of mitochondrial ROS was less important in platelets [51]. This may explain why there were no differences in cytosolic ROS levels between Sirt3-/- and WT (Fig 3C). Platelet aggregation was augmented only when cells were stimulated with low concentration of collagen. Additionally, there was no difference in thrombin-induced surface expression of P-selectin and activation of GPIIbШa between the genotypes. It can be concluded that deficiency of Sirt3 did not significantly impact platelet function. In contrast, platelets from NOX2-/- mice showed more than a 50% reduction in thrombin-induced ROS generation, resulting in less arterial thrombosis [52]. It is well known that exogenous ROS enhances platelet adhesion and aggregation. However, the stimuli that were required to accelerate platelet aggregation were too severe to achieve this endogenously [53, 54]. In conclusion, the elevation of mitochondrial ROS observed in platelets from Sirt3-/- mice was not strong enough to augment platelet aggregation, and thus, did not impact venous thrombosis.

According to the significant reduction in the circulating platelet counts upon onset of thrombosis in Sirt3-/- mice (Fig 5G), it can be speculated that blood cells or endothelium responsible for platelet adhesion and aggregation are slightly more activated during IVC stenosis than in WT mice. Also, the elevation of neutrophil counts in Sirt3-/- mice after IVC stenosis raises the possibility that neutrophils were recruited from marginated pools in the bone marrow or spleen in response to thromboinflammation [55], and were recruited faster in Sirt3-/- than in WT.

Sirt3 deficiency inactivates MnSOD [22], and while MnSOD deficiency is lethal in mice [56, 57], MnSOD+/- mice show dysfunction of endothelial relaxation with elevated vascular ROS [58, 59]. In the DVT model, mild activation of endothelial cells in Sirt3-/- may result in enhanced platelet adhesion to endothelial cells, leading to consumption of circulating platelets, and hence, a reduction of platelet counts [60]. In aged mice, inactivation of endothelial MnSOD could be more prominent, resulting in dysfunction of endothelium [58, 60]. However, aged mice were not investigated in the present study, as accurate IVC stenosis is hindered by the deposition of fatty tissue around vessels. Moreover, diseases known to prevail with age occur at an accelerated pace in Sirt3 KO mice [29]. Platelet- or neutrophil-specific Sirt3-deficient mice would therefore be best suited to address the role of Sirt3 in platelet and neutrophil function in aged animals.

Our results indicate that both mouse neutrophils and platelets express Sirt3, although their physiological responses are not exacerbated in the absence of Sirt3. We have shown that deficiency of Sirt3 augmented ROS generation in the mitochondria with stimulation. However, we did not see detrimental effects in Sirt3-/- mice in a DVT model. In conclusion, Sirt3 deficiency does not impact NET formation and platelet functions in healthy young mice.

## Supporting information

S1 Fig Supporting information for Western blotting. (A) Western blot analysis using bone marrow neutrophil lysates from Sirt3-/- mice or WT mice. Neutrophil lysate sample was blotted with Sirt3 antibody, then re-probed with GAPDH antibody. (B) Western blot analysis using washed platelet lysates from Sirt3-/- mice or WT mice. Platelet lysate sample was blotted with Sirt3 antibody, then re-probed with β-Tubulin antibody.
(TIF)

S2 Fig Supporting information for platelet serotonin release. Released serotonin was measured in the supernatant of unstimulated (unst.), thrombin- or collagen-activated platelets. Total serotonin content was quantified in platelet lysates. Results are presented as mean ± SD. WT n = 4, Sirt3-/- n = 4 for unstimulated, collagen- or thrombin-activated platelets; total platelet serotonin content: WT n = 3, Sirt3-/- n = 4.
(TIF)
