# Supplementary figures and images for: Sirt3 deficiency does not affect venous thrombosis or NETosis despite mild elevation of intracellular ROS in platelets and neutrophils in mice
Source: PLoS One. 2017 Dec 13;12(12):e0188341. doi: 10.1371/journal.pone.0188341 (PMC5728566; doi:10.1371/journal.pone.0188341)

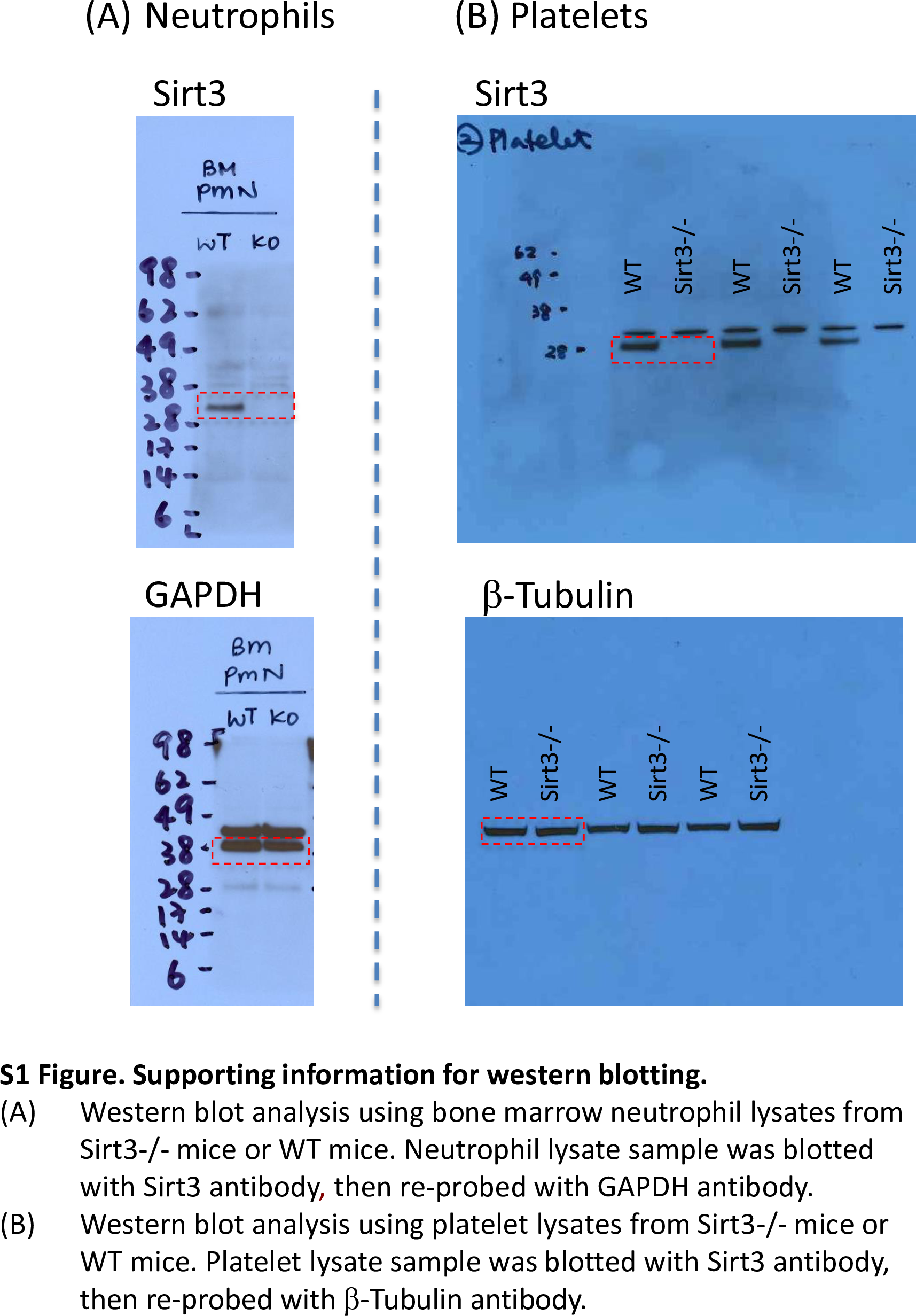

Supplement: S1 Fig — (A) Western blot analysis using bone marrow neutrophil lysates from Sirt3-/- mice or WT mice. Neutrophil lysate sample was blotted with Sirt3 antibody, then re-probed with GAPDH antibody. (B) Western blot analysis using washed platelet lysates from Sirt3-/- mice or WT mice. Platelet lysate sample was blotted with Sirt3 antibody, then re-probed with β-Tubulin antibody. (TIF) [file pone.0188341.s001.tif]

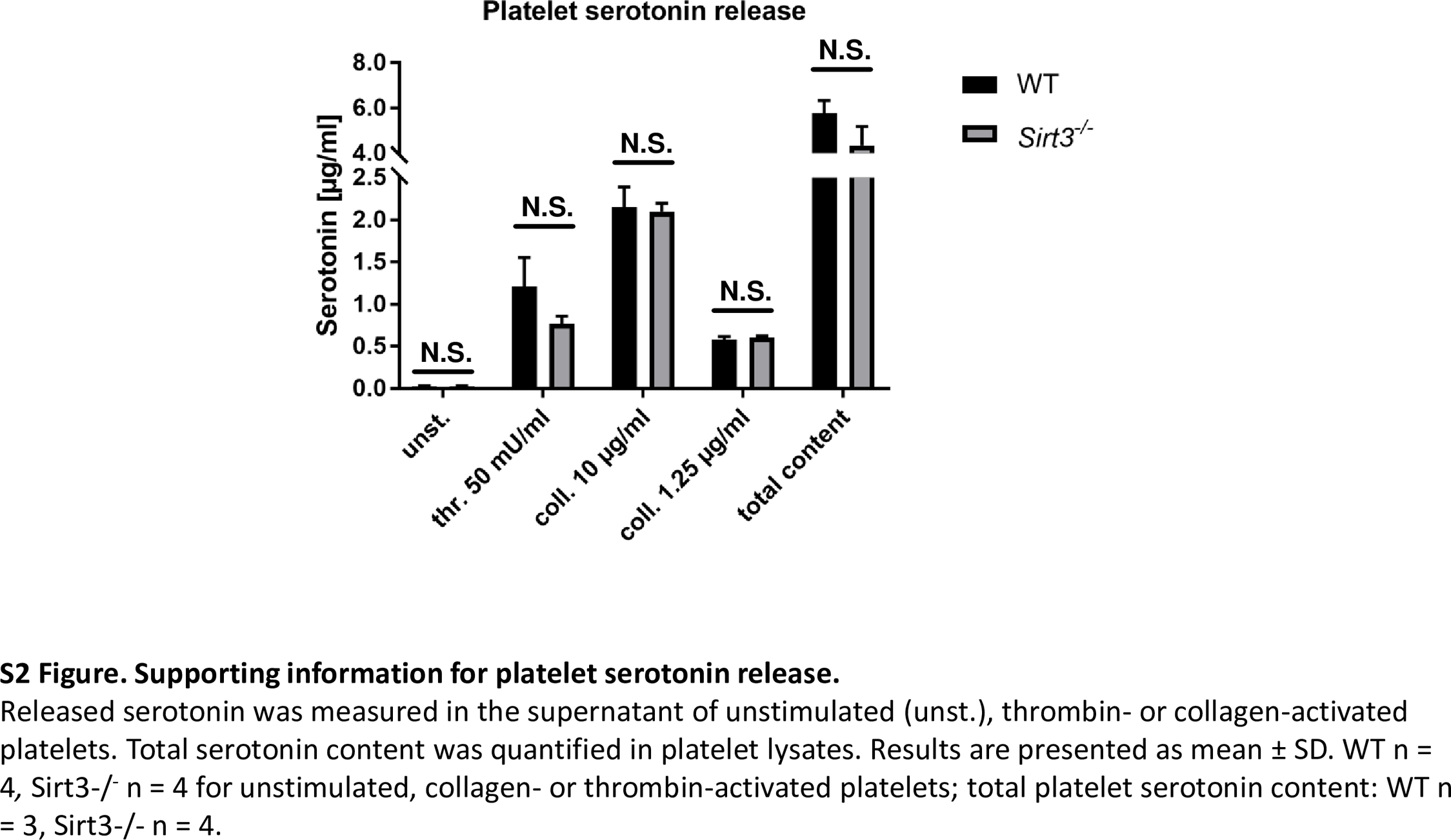

Supplement: S2 Fig — Released serotonin was measured in the supernatant of unstimulated (unst.), thrombin- or collagen-activated platelets. Total serotonin content was quantified in platelet lysates. Results are presented as mean ± SD. WT n = 4, Sirt3-/- n = 4 for unstimulated, collagen- or thrombin-activated platelets; total platelet serotonin content: WT n = 3, Sirt3-/- n = 4. (TIF) [file pone.0188341.s002.tif]
